# Store-operated Ca^2+^ entry regulatory factor alters murine metabolic state in an age-dependent manner via hypothalamic pathways

**DOI:** 10.1093/pnasnexus/pgad068

**Published:** 2023-03-04

**Authors:** Diana Gataulin, Yael Kuperman, Michael Tsoory, Inbal E Biton, Tomer Nataniel, Raz Palty, Izhar Karbat, Anna Meshcheriakova, Eitan Reuveny

**Affiliations:** Department of Biomolecular Sciences, Weizmann Institute of Science, Rehovot 760001, Israel; Department of Veterinary Resources, Weizmann Institute of Science, Rehovot 760001, Israel; Department of Veterinary Resources, Weizmann Institute of Science, Rehovot 760001, Israel; Department of Veterinary Resources, Weizmann Institute of Science, Rehovot 760001, Israel; Department of Biochemistry, Rappaport Faculty of Medicine, Technion, Haifa 31096, Israel; Department of Biochemistry, Rappaport Faculty of Medicine, Technion, Haifa 31096, Israel; Department of Biomolecular Sciences, Weizmann Institute of Science, Rehovot 760001, Israel; Department of Biomolecular Sciences, Weizmann Institute of Science, Rehovot 760001, Israel; Department of Biomolecular Sciences, Weizmann Institute of Science, Rehovot 760001, Israel; Department of Veterinary Resources, Weizmann Institute of Science, Rehovot 760001, Israel; Department of Molecular Neuroscience, Weizmann Institute of Science, Rehovot 760001, Israel

## Abstract

Store-operated calcium entry (SOCE) is a vital process aimed at refilling cellular internal Ca^2+^ stores and a primary cellular signaling driver for transcription factors’ entry to the nucleus. SOCE-associated regulatory factor (SARAF)/TMEM66 is an endoplasmic reticulum (ER)-resident transmembrane protein that promotes SOCE inactivation and prevents Ca^2+^ overfilling of the cell. Here, we demonstrate that mice deficient in SARAF develop age-dependent sarcopenic obesity with decreased energy expenditure, lean mass, and locomotion without affecting food consumption. Moreover, SARAF ablation reduces hippocampal proliferation, modulates the activity of the hypothalamus–pituitary–adrenal (HPA) axis, and mediates changes in anxiety-related behaviors. Interestingly, selective SARAF ablation in the hypothalamus's paraventricular nucleus (PVN) neurons reduces old age-induced obesity and preserves locomotor activity, lean mass, and energy expenditure, suggesting a possible central control with a site-specific role for SARAF. At the cellular level, SARAF ablation in hepatocytes leads to elevated SOCE, elevated vasopressin-induced Ca^2+^ oscillations, and an increased mitochondrial spare respiratory capacity (SPC), thus providing insights into the cellular mechanisms that may affect the global phenotypes. These effects may be mediated via the liver X receptor (LXR) and IL-1 signaling metabolic regulators explicitly altered in SARAF ablated cells. In short, our work supports both central and peripheral roles of SARAF in regulating metabolic, behavioral, and cellular responses.

**Significance Statement**

Overweight and obesity are significant medical problems that affect close to two billion people worldwide and are considered a twenty-first-century pandemic. Multiple factors can lead to their pathologies. Here, we show that mice lacking SARAF display age-dependent symptoms resembling sarcopenic obesity, including decreased metabolic rate, lean mass, and locomotion, without affecting food consumption. At the cellular level, SARAF ablation increases SOCE, elevates Ca^2+^ oscillation in response to vasopressin, and increases the mitochondria's SRC, lipid droplet hypertrophy, brown adipose tissue whitening, and hepatic steatosis. Selective removal of SARAF from PVN neurons has opposite phenotypes. These results provide a unique link between Ca^2+^ homeostasis regulation abnormalities and obesity, mainly in old age, and provide a hint for central control of this process.

## Introduction

Overweight and obesity affect almost 2 billion people worldwide [as calculated by the World Health Organization (WHO) in 2016] and are considered a twenty-first-century pandemic. Diet and exercise are the primary prevention and treatment strategies (https://www.who.int/health-topics/obesity#tab=tab_1). However, understanding the genetic factors involved in the central circuitry, metabolism, and adipose tissue homeostasis may improve the ability to treat obesity with precision (1, 2). Metabolic homeostasis of the entire organism is a balanced feedback mechanism involving both the central nervous system (CNS) and periphery, where the hypothalamic paraventricular nucleus (PVN) plays a central role (3); it, directly and indirectly, regulates critical hormones such as corticosterone (cortisol in humans), adrenaline, vasopressin, oxytocin, and thyrotropin-releasing hormone (TRH) (4), which control the body's metabolic state (5). Many intracellular pathways respond to these signaling cues to affect metabolic homeostasis, where changes in intracellular Ca^2+^ levels are central (6–8). It is thus critical to have tight regulation of intracellular and intraorganelle Ca^2+^ levels. This task is a well-coordinated action involving many proteins that either pump Ca^2+^ out from the cytosol or into intracellular organelles such as mitochondria and the endoplasmic reticulum (ER) (8).

Store-operated calcium entry (SOCE) is one of several processes that participate in the cell's Ca^2+^ homeostasis (9). In most cell types, it replenishes the Ca^2+^ cellular stores, like the ER and mitochondria. In some, it also plays a crucial role in transcription factors’ entry to the nucleus to activate gene transcription (10). The activation of SOCE follows the release of Ca^2+^ from ER stores by inositol triphosphate (IP_3_), the product of the breakdown of phosphatidylinositol diphosphate (PIP2) by PLCβ or PLCγ activation via G_q_/11-coupled G protein-linked receptors, or by receptor tyrosine kinases, respectively (11, 12). SOCE activity depends on two proteins, a stromal interaction molecule (STIM), an ER transmembrane protein with a Ca^2+^-sensing domain at its ER luminal side, and a plasma membrane (PM)-resident Ca^2+^ channel (Orai) (13–17). SOCE is triggered by the depletion of Ca^2+^ from the ER lumen, oligomerization of STIM, and activation of Orai at PM–ER junctions, allowing the flow of Ca^2+^ ions into the cell to replenish the depleted stores. An additional layer of SOCE activity involves STIM and Orai interactions with the transient receptor potential channels (TRPCs) and nonselective cation channels (18, 19).

Calcium signaling involving the ER and mitochondria is compromised in obesity and metabolic diseases (6, 20). SOCE is a crucial element in lipid metabolism and adiposity; specifically, it plays a role in the mobilization of fatty acids from lipid droplets, lipolysis, and mitochondrial fatty acid oxidation (21, 22). SOCE is also necessary for glucose-stimulated pancreatic insulin secretion (23, 24). Moreover, Ca^2+^ signaling and SOCE are involved in proper liver function, including bile secretion, proliferation, oscillatory response to hormones, cholesterol, and glucose metabolism (25, 26). Alterations in liver Ca^2+^ homeostasis are associated with ER stress, inflammation, impaired insulin function, and abnormal glucose metabolism (27, 28). Furthermore, SOCE activity was found to be impaired in the liver of obese murine models (29, 30). SOCE was also implicated in myocytes’ function, specifically its importance in the proper operation of the sarcoplasmic reticulum (31–33), and its activity changes with age (34, 35).

SARAF is an ER-resident protein that was shown to associate with STIM and promote SOCE inactivation (36–39). SARAF plays a key role in shaping cytosolic Ca^2+^ signals and determining the content of the major intracellular Ca^2+^ stores, which is probably important in protecting the cell from Ca^2+^ overfilling. SARAF is localized to PIP2-rich PM junctions (40) and is activated by dimerization at its ER luminal end (41) when stores are full by a still unknown mechanism. Reduced SARAF levels increase intracellular SOCE and were recently found to be involved in several physiological and pathological conditions (42–44); for review, see (45). Moreover, SARAF is an androgen-responsive marker for prostate cancer and mTOR-dependent cardiac growth (46, 47). Here, we report on the consequences of knocking out SARAF globally and in a PVN-specific manner in mice. Knockout (KO) of SARAF introduces a new role for SARAF in a physiological context. SARAF-KO mice gain weight and lose lean mass at a later age. This weight gain is not the consequence of increased food intake but is dependent on PVN-associated circuits. At the cellular level, SARAF increases mitochondrial respiration in addition to its expected reduction of SOCE activity. This report may place SARAF as an important component in the pathophysiology of obesity.

## Results and discussion

### Generation of SARAF-KO mice

**Fig. 1.**
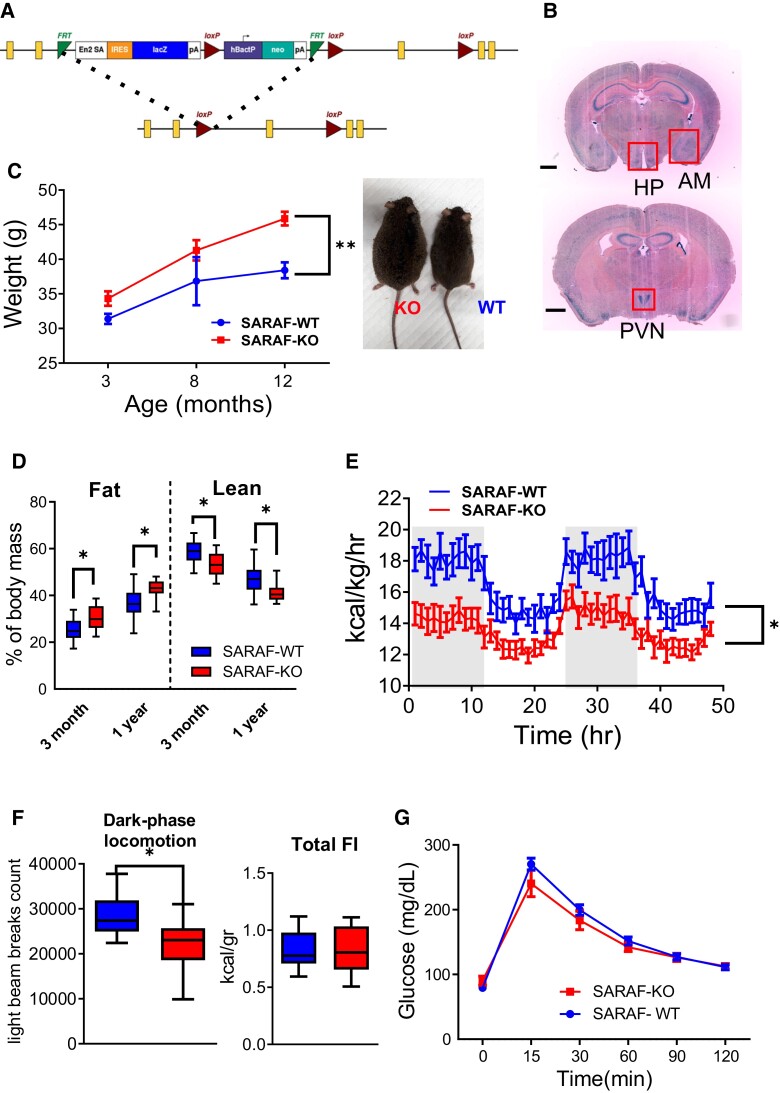
Generation of SARAF conditional mice, SARAF expression in the brain, and SARAF^fl/fl^ PGK-Cre^+^ metabolic phenotype. A) Schematic representation of the SARAF knock-in cassette and after flipase exertion of the cassette leaving crucial exon 3 flanked by loxP sites. B) X-gal-stained coronal brain sections of KOMP cassette-inserted heterozygous mice, expressing β-gal at the sites of SARAF expression (scale bar: 1 mm) (see magnified images in Fig. S5). C) Changes in body weight of SARAF-WT (*n* = 13) and SARAF-KO (*n* = 11) over time. Inset, representative photos of SARAF-WT (right) and SARAF-KO (left) mice at 1 year old. D) Lean and fat mass percentage of SARAF-WT (*n* = 13), and SARAF-KO (*n* = 11) mice at 3 months and 1 year old. E and F) PhenoMaster calorimetry metabolic analysis of 3-month-old mice (SARAF-WT, *n* = 8; SARAF-KO, *n* = 10). E. Heat production over time. F) Dark-phase locomotion. G) Total food intake. H) Three-month-old mice glucose tolerance test (SARAF-WT, *n* = 7; SARAF-KO, *n* = 9).

To study the physiological role of SARAF in the whole animal context, we utilized the knockout-first allele gene trap KOMP repository to generate, first, LacZ-reporting animals followed by the generations of SARAF conditional knockout animals, termed SARAF^fl/fl^, as described in the experimental procedure section and graphical representation (Fig. 1A). SARAF, although ubiquitously expressed, is highly expressed in the immune and neuronal tissues (36); we focused on its expression in the brain via LacZ staining and found it has a marked expression in the hippocampus and hypothalamus (specifically the PVN) and the amygdala (Fig. 1B). A whole-body knockout line was generated by crossing SARAF^fl/fl^ mice with transgenic animals ubiquitously expressing the PGK promoter-driven Cre recombinase to generate PGK-Cre^+^:SARAF^fl/fl^, termed SARAF-KO. The knockout of SARAF was validated via Western blotting of the brain tissue and compared with their littermates SARAF^fl/fl^PGK-Cre^−^, who were homozygous to the floxed SARAF, but did not express Cre recombinase, termed SARAF-WT (Figr. S1A). SARAF-KO mice were viable and bred normally following Mendelian distribution.

### SARAF-KO mice have impaired metabolic function and lipid accumulation

Male SARAF-KO mice were compared with their WT littermates and displayed a significant increase in body weight. The weight differences were significantly higher from early adulthood (3 months), where the SARAF-KO mice weight was, on average, about 10% more than that of SARAF-WT mice. The weight differences increased with age. At 1 year old, SARAF-KO mice weigh nearly 20% more than the WT mice (Fig. 1C). Linear growth was not altered in the SARAF-KO mice (Supp.1B). Interestingly, the weight differences were also reflected in body composition alterations, including an increased percentage of fat mass and a lower percentage of lean mass in the SARAF-KO mice, suggesting a possible role for SARAF in mediating sarcopenic obesity-like symptoms (Fig. 1D). Indirect calorimetry assessment of SARAF-KO mice revealed a lower metabolic rate (as manifested in heat production) and reduced locomotion (measured during the active phase in the diurnal cycle) (Fig. 1E and F). However, the abovementioned changes were not associated with significant differences in food intake (Fig. S1C). Furthermore, food intake after 5 hours of fasting, in a refeed experiment, did not alter either the weight loss or the weight gain (Fig. S1D). Interestingly, fasting glucose levels and the response to glucose load (glucose tolerance test, GTT) were normal in the SARAF-KO mice (Fig. 1G).

**Fig. 2.**
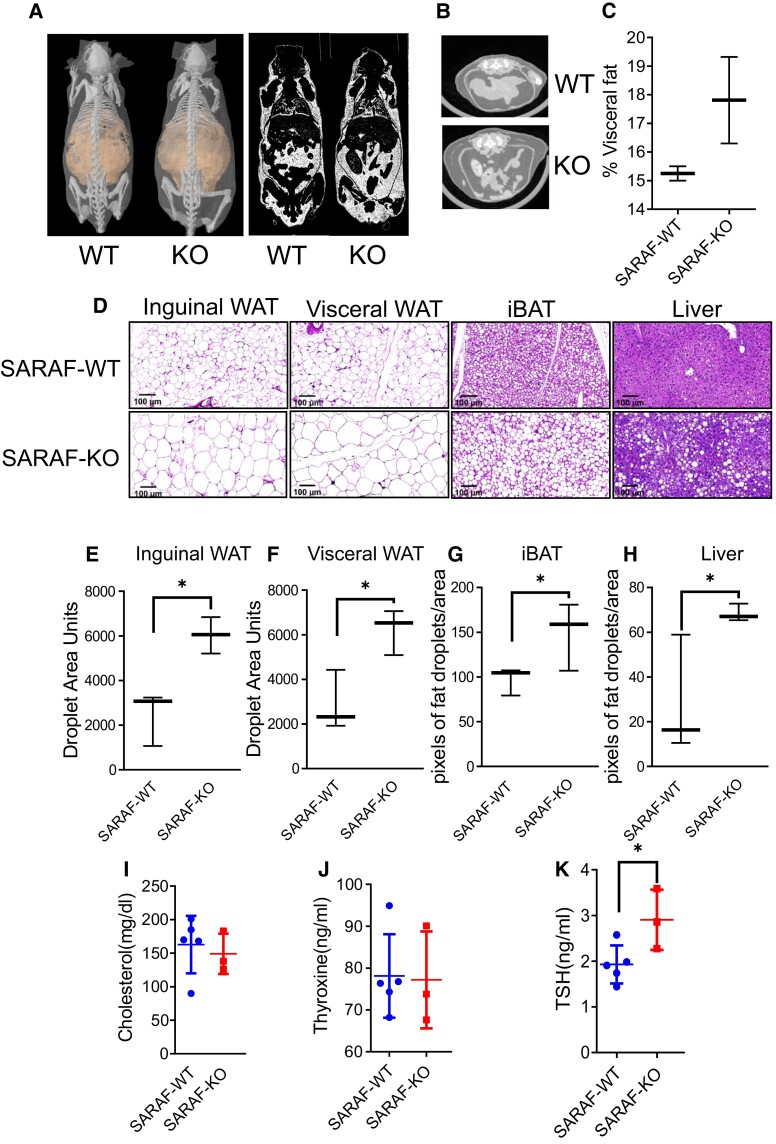
Characterization of SARAF-KO lipid deposition phenotype and voluntary training treatment. A) Micro-CT imaging and whole-body fat (right) distribution analysis of representative 1-year-old SARAF-WT and SARAF-KO mice. B) Abdominal fat representative images of CT images of abovementioned mice. C) Quantification of visceral fat deposition. D) Inguinal WAT, visceral WAT, liver, and iBAT tissue histology in 1-year-old SARAF-WT and SARAF-KO mice. Size bar: 100 μm. E and F) Inguinal and visceral WAT droplet size quantification. G and H) iBAT and liver pixels of fat droplets/area quantification. I) Serum analysis of cholesterol levels of 1-year-old mice. J) Serum analysis of thyroxine levels of 1-year-old mice. K) Serum analysis of TSH levels of 1-year-old mice.

We then examined the adipose tissue distribution in the elderly SARAF-KO mice using computed tomography (CT) imaging to reveal that the excess fat was distributed throughout the body with a high tendency for abdominal accumulation (Fig. 2A–C). Hematoxylin and eosin (H&E)-stained white adipose tissue (WAT) droplet size analysis of inguinal and visceral adipose tissues (iWAT and vWAT, respectively) further revealed significant hypertrophy of fat cells. Moreover, we found that the fat accumulated both in the intracapsular brown adipose tissue (iBAT) and in the liver, resulting in iBAT whitening and hepatic steatosis (Fig. 2D–H). These histological phenotypes were seen in 3-month-old mice and an upsurge in 1-year-old mice, except for liver fat accumulation, which is not apparent yet in younger mice (Supp.1E-I). The BAT is an organ that contributes to systemic metabolic homeostasis and thermoregulation, and its size is associated with various pathologic conditions, including obesity (48, 49). Interestingly, iBAT whitening can be prevented by inhibiting Ca^2+^ overload in the mitochondria (50). Hepatic steatosis is accompanied by cellular Ca^2+^ imbalance and is a predisposition for a nonalcoholic fatty liver disease that might lead to liver cirrhosis and cancer (51). The weight- and fat-associated changes in elderly SARAF-KO mice were also accompanied by subclinical hypothyroidism, manifested by normal serum levels of thyroxine and cholesterol and elevated TSH levels (52) (Fig. 2I–K). Subclinical elevation of TSH might influence resting metabolic rate and hint at early thyroid dysfunction (53). The correlation between the old age increase in TSH and hepatic steatosis may raise the possibility of a thyroid–liver interaction (54).

### SARAF ablation in SIM1 neurons improves metabolic function and hints toward hypothalamic metabolic feedback

**Fig. 3.**
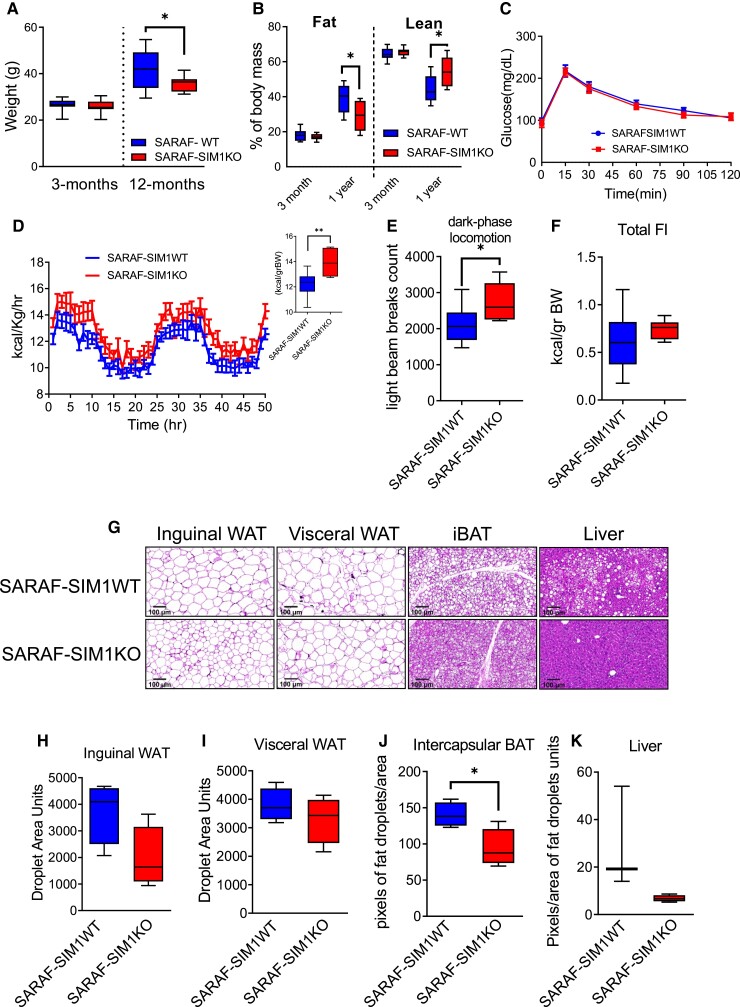
SARAF-SIM1KO mice metabolic-related phenotypes. A) Three-month and 1-year-old SARAF-SIM1WT (*n* = 16) and SARAF-SIM1KO (*n* = 9) mice body weight comparison. B) Lean and fat mass percentage of SARAF-SIM1WT and SARAF-SIM1KO 3-month and 1-year-old mice of the same mice as in A. C) Three-month-old glucose tolerance test (SARAF-SIM1WT, *n* = 11; SARAF-SIM1KO, *n* = 6). D–F) PhenoMaster calorimetry metabolic analysis of 1-year-old mice (SARAF-SIM1WT, *n* = 10; SARAF-SIM1KO, *n* = 6). D) Heat production over time and heat production per body weight (inset). E) Dark-phase locomotion. F) Total food intake. G) Inguinal WAT, visceral WAT, liver, and iBAT tissue histology in 1-year-old SARAF-SIM1WT and SARAF-SIM1KO mice. Size bar: 100 μm. H and I) Inguinal and visceral WAT droplet size quantification. J and K) iBAT and liver pixels of fat droplets/area quantification.

SARAF-regulated metabolic phenotypes discussed above could stem from several metabolic organs, including adipose-, muscle-, and brain-derived regulation. Because of the marked SARAF expression in the hypothalamus, we chose to focus on the role of hypothalamic SARAF in energy homeostasis. To this end, we crossed the SARAF^fl/fl^ mice to SIM1 promoter-driven Cre recombinase-expressing mice (SIM1-Cre) (55) to induce SARAF deletion specifically in the hypothalamic PVN, termed SARAF-SIM1KO. Allele recombination was validated via site-specific punch-PCR of the PVN (Suppl. 2A). Unlike SARAF-KO, 3-month-old SARAF-SIM1KO mice had similar body weights and composition to their WT littermates. However, at 1 year old, these mice were found to have improved age-related metabolic phenotypes, inversely mirroring the whole-body SARAF-KO. At 1 year old, SARAF-SIM1KO mice had lower body weight, reduced fat percentage, and increased lean mass percentage compared to SARAF-SIM1WT littermates (Fig. 3A and B). Like the whole-body knocked-out mice, SARAF-SIM1KO mice did not have an altered response to glucose or differences in food consumption, either under basal or refeed-challenged conditions (Fig. 3C and Supp. 2B-C). Moreover, their heat production and locomotor activity were elevated with an opposite tendency to the SARAF-KO mice (Fig. 3D–F). Those phenotypes are apparent only at old age (over 1 year old) except for increased locomotion, which was already evident in 3-month-old mice (Fig. S2D–F). The increased locomotion in SARAF-SIM1KO mice is of interest since it is consistent from a young age to old age and thus may drive the phenotype by increasing lean mass and/or changing energy expenditure. Several studies have implicated hypothalamic PVN in the regulation of locomotion (56–58). Interestingly, when examining lipid accumulation in the SARAF-SIM1KO mice, we noticed that the BAT had reduced fat accumulation compared with the wild-type littermates. We did not witness differences in WAT droplet size or fat accumulation in the liver (Fig. 3G–K). When challenging these mice with a diet rich in fat and carbohydrates (Western diet) for 18 weeks, their total increases in body weight, composition, and calorimetry analysis were similar between the two groups (Fig. S3A–E). In Western diet-fed mice, lipid accumulation phenotype, glucose tolerance, and refeed responses were also unchanged (Fig. S3E–J), thus strengthening the assumption that SARAF-related metabolic phenotypes in SARAF-SIM1KO mice are not related to food intake-related mechanism. These results point toward coordinating the BAT and thermogenesis by the PVN via the Ca^2+^ homeostasis mechanism (5). SARAF-SIM1KO mice did not exhibit altered anxiety-related behavioral phenotypes, suggesting that SARAF, although ubiquitously expressed, plays a site-specific role (Fig. S4G–I).

A recent study examined the involvement of SOCE in another subset of hypothalamic neurons, Agouti-related peptide (AgRP)-producing neurons (59). In this study, Chen et al. demonstrated that with specific ablation of STIM1 from these neurons, the mice displayed reduced appetite and increased heat production, associated with increased oxygen consumption. The opposite phenotypes were displayed when a constitutively active STIM1 mutant was introduced via viral infection to these neurons. This study underlines the complex regulation of feeding behavior, especially in light of the differential dependence of fat accumulation on the type of food consumed. The picture is even more complex when considering our results with SARAF-KO (increased SOCE activity), which shows that weight gain is not associated with disturbed satiety (Suppl. 1C and 2B). AgRP neurons negatively regulate the PVH that contains both MC4R and prodynorphin-expressing neurons (60). On the other hand, AgRP neurons are negatively regulated by kisspeptin neurons via metabotropic glutamate transmission, and deletion of STIM1 from these neurons protects mice from developing obesity and glucose intolerance with high-fat dieting (61, 62). In the hypothalamic arcuate–median eminence region, SIM1-expressing neurons neither express AgRP nor MC4R but do express STIM1 and SARAF (63) (Fig. S7, see also https://singlecell.broadinstitute.org/single_cell). Conversely, neurons that do not express SIM1 co-express AgRP, MC4R SARAF, and STIM1 to different degrees. This differential expression profile, and the intricate neuronal circuit controlling energy metabolism, may provide a clue into the opposite effect of SARAF-KO vs. SARAF-SIM1KO sarcopenic-like obesity and energy expenditure-observed phenotypes.

### SARAF ablation decreases hippocampal proliferation and affects the stress response and HPA axis activation

**Fig. 4.**
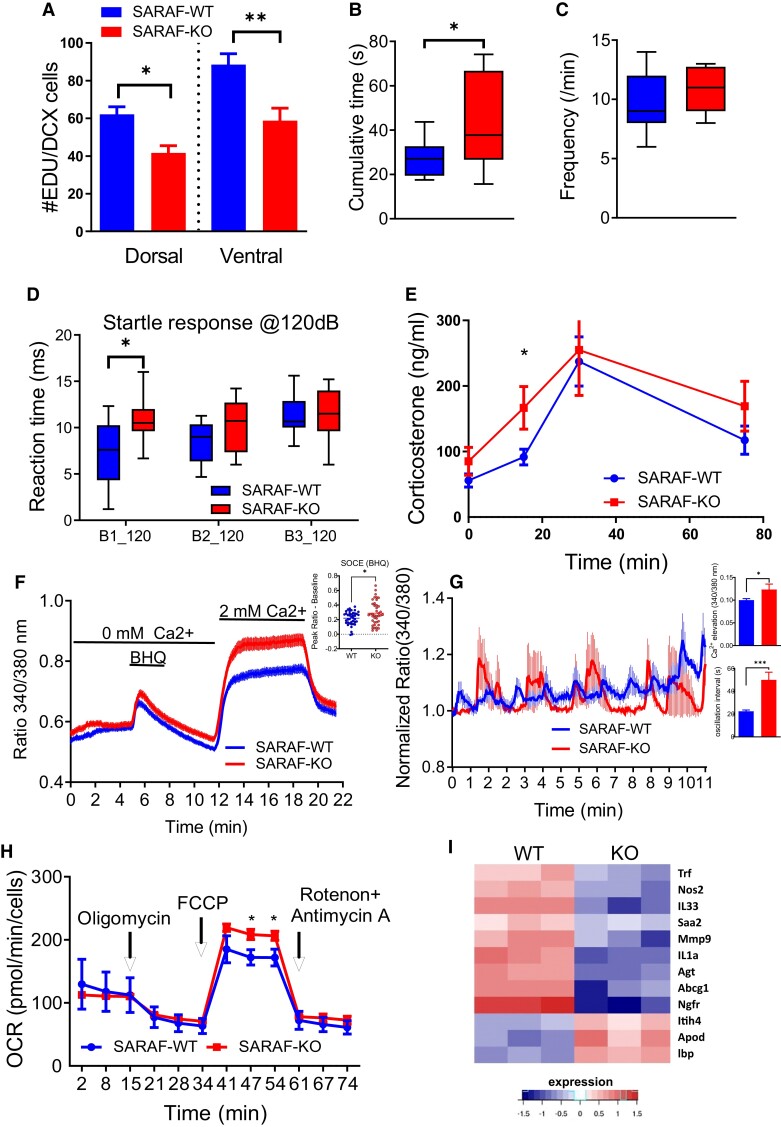
Hippocampal neurons proliferation, anxiety-related behavioral phenotypes, and hepatocyte cellular phenotypes of SARAF^fl/fl^PGK-Cre^+^ mice. A) EDU (5-ethynyl-2´-deoxyuridine) proliferation analysis of SARAF-WT and SARAF-KO mice at the dorsal and ventral hippocampal dentate gyrus proliferation quantification. B and C) Three-month-old SARAF-WT (*n* = 11) and SARAF-KO (*n* = 10) mice performance in anxiety-related tests: B) dark–light transfer. C) Frequency of exits. D) ASR test. Reaction time in three blocks of 120-db stimuli. E) Blood corticosterone levels following 15-min restrain stress (SARAF-WT, *n* = 8; SARAF-KO, *n* = 7). F) Fura-2 AM Ca^2+^ imaging trace of primary hepatocytes extracted from SARAF-WT and SARAF-KO mice and the quantification of SOCE levels (inset). G) Ca^2 +^ oscillations in hepatocytes induced by vasopressin (1 nM) as measured by FURA-2 AM. Quantification of Ca^2+^ elevation and oscillation interval (inset). H) Representative experiment of mitochondrial respiration of SARAF-WT (*n* = 3) and SARAF-KO (*n* = 3) primary hepatocytes and their SRC. I) Heatmap representation of LXR/RXR hepatic (liver X receptor) pathway activation which was indicated by ingenuity platform in MEF RNA sequencing results.

SARAF was highly expressed in the hippocampus (Fig. 1B) and specifically in a subset of doublecortin (DCX)-positive neuronal progenitors, indicative of proliferating neural stem cells (Fig. S4A). Since hippocampal proliferation has a marked impact on anxiety and metabolism via the HPA axis (64, 65), we sought to examine the impact of SARAF on hippocampal proliferation and its effect on the HPA axis. The altered function of this system may affect the metabolic phenotype and may account, in part, for the phenotypes reported above. EdU (5-ethynyl-2′-deoxyuridine) incorporation was used to examine hippocampal cell proliferation in the SARAF-WT and the SARAF-KO mice. EDU incorporation assay revealed a significant decrease in proliferation in the dorsal and the ventral hippocampus dentate gyrus in the SARAF-KO mice (Fig. 4A). This decrease was independently validated via immunostaining against proliferating cell nuclear antigen (PCNA) as the proliferation marker (Fig. S4B) and repeated in primary mouse embryonic fibroblast (MEF) cultures derived from SARAF-WT and SARAF-KO using staining for Ki67 expression (Fig. S4C).

Behaviorally, SARAF-KO mice exhibited decreased anxiety-like behavior as manifested in the dark–light transfer (DLT) by increased time spent in the lit compartment (Fig. 4B and C) and in the acoustic startle response (ASR) experiment, where they exhibited a longer reaction time in the first set of stimulus presentations (Fig. 4D). Counterintuitively, SARAF-KO knocked-out mice exhibited a mild increase in HPA axis activation, as suggested by a more robust immediate corticosterone response to restraint stress (Fig. 4E). This discrepancy may stem from differences in the timing of the assessment, 5–10 min the initiation of the exposure to stress in the DLT and ASR tests, as opposed to about 30 min in the CORT assessment. Interestingly, despite the reduced neurogenesis observed, long-term memory, assessed using the Morris water maze (Fig. S4F), and short-term memory, assessed using the Y-maze (Fig. S4D and E), were not altered.

### SARAF ablation leads to increased cellular SOCE, higher mitochondrial spare respiratory capacity, and altered gene expression patterns

Cellular metabolic functions influence global metabolic phenotypes and behavior; specifically, mitochondrial respiration greatly influences global metabolic phenotypes (66). Moreover, since the hypothalamus, directly via neuronal connections and indirectly via hormonal secretion, regulates metabolic organs (67, 68), we sought to examine whether the cellular metabolic functions were consistent with the global phenotypes we observed in the KO animals. More specifically, the liver is tightly regulated by PVN innervation and hormonal regulation (69, 70). For this reason, we extracted primary hepatocytes from SARAF-KO mice and examined their Ca^2+^ signaling and SOCE by FURA-2 AM-based Ca^2+^ imaging. We induced SOCE by the transient sarco/endoplasmic reticulum Ca^2+^-ATPase (SERCA) inhibitor BHQ. SOCE activity was elevated in the knocked-out hepatocytes, confirming our previous in vitro experiments (36). Hepatocyte SOCE was inhibited by the Orai inhibitors La^3+^ or 2-ABP, therefore hinting at the involvement of the classical SOCE machinery (71) (Fig. 4F).

Ca^2+^ oscillations are a significant driver of cellular signaling, and they are mediated by SOCE and by mitochondrial Ca^2+^ uptake (72). Vasopressin is a pituitary-secreted hormone that regulates several physiological functions, including behavior, thermoregulation, water absorption, liver function, and adipogenesis (73, 74). When examining Ca^2+^ oscillations triggered by physiological levels of vasopressin (1 nM), we observed a marked increase in release amplitude and an increase in oscillation intervals in the SARAF-KO-derived hepatocytes (Fig. 4G). The latter strengthens the idea that SARAF may affect hypothalamic control over metabolic organs, including the liver. Next, we sought to examine cellular metabolic function by measuring mitochondrial respiration by directly examining the cellular oxygen consumption rate (OCR) (75). This function is highly influenced by the cellular Ca^2+^ levels and influences global metabolic phenotypes (66, 76, 77). Cellular mitochondrial respiration was markedly altered in SARAF knocked-out hepatocytes, having a significantly higher spare respiratory capacity (SRC) function (Fig. 4H). These findings suggest that SARAF, via the modulation of SOCE activity, has a great influence on metabolic organs at a cellular level, like the liver, with altered mitochondrial metabolism and SOCE. Since Ca^2+^, in addition to its direct regulatory action, can affect, at longer time scales, gene transcription, we set to examine gene transcription patterns by RNA sequencing embryonic fibroblast (MEF) cells derived from SARAF-WT and SARAF-KO animals. We analyzed the results for canonical pathway enrichment using QIAGEN's Ingenuity software and identified that LXR (liver X receptor)/RXR (retinoid X receptor) signaling pathway was significantly altered (Fig. 4I), hinting, again, at SARAF importance in proper liver function. The LXR/RXR signaling pathway genes that were altered include IL1α, IL33, LBP, and ApoD, all of which have indications for metabolic function (78, 79). LXR/RXR and the related LXR-regulated IL-1-signaling pathways were previously shown to influence metabolism via the hypothalamus and directly influence adipose tissue and the liver (80–82).

## Conclusion

This study demonstrates that SARAF is involved in the physiological regulation of age-dependent metabolic rate (as manifested by energy expenditure) and locomotion without affecting food consumption or clear blood glucose handling. Moreover, SARAF was involved in a complex metabolic phenotype that regulates lipid metabolism in the adipose tissue, the BAT, and the liver. These global phenotypes are accompanied by SARAF-mediated cellular metabolic functions, including hepatocyte's SOCE, vasopressin-evoked Ca^2+^ oscillations, and mitochondria's SRC. Examining SARAF's contribution to the CNS regulation functions indicated its involvement in PVN-regulated autonomic–sympathetic control of energy expenditure, locomotion, and BAT profile. Moreover, SARAF was found to regulate hippocampal proliferation while having some effects on the HPA axis and anxiety-related behavior.

Cellular Ca^2+^ balance is a significant factor in fine-tuning metabolic function in different organs, including the liver, muscle, adipose tissues, and neurons; even slight changes in Ca^2+^ levels might affect their function (83, 84). SARAF is expressed in the Sim-1-expressing neurons of the brain, the muscle, and the BAT. SOCE homeostasis, regulated by SARAF, is especially important in a subset of specific cells/tissues described in this study; it is suggested, however, that additional subtle and yet-to-be-discovered SARAF-related physiological functions are probably affected as well. Moreover, other proteins essential for maintaining Ca^2+^ homeostasis, like SERCA, whose activity is reduced with aging (85), may be essential in the absence of SARAF. In this study, the influence of SARAF on SRC was indicated in the hepatocytes’ OCR measurements, underlining the importance of maintaining appropriate Ca^2+^ levels for healthy cell function (86). Interestingly, SRC is a known influencer of aging muscle function and a driver of sarcopenia (87). Thus, SRC might drive SARAF's involvement in lean mass maintenance. In the liver, SARAF causes improved SRC, seemingly opposite to the previously described papers; however, an increase in SRC is possible in case of damaged oxidative phosphorylation as indicated in some reports (88). Interestingly, running wheel data show that training increases in aged muscles (89). Furthermore, SRC is also elevated in the hyperglycemic adipocytes, allowing for fast adaptation and recovery. SARAF ablation in the metabolic organ forces increased SRC levels as an adaptive response to less than favorable conditions (90).

SARAF's role in hippocampal neurons needs further refining; specifically, it is perplexing that SARAF's involvement in hippocampal proliferation did not alter learning and memory. Nevertheless, it has been previously discussed that significant cellular aberration should occur to influence a behavioral phenotype (91). Given the importance of Ca^2+^ regulation for cellular physiology, compensatory mechanisms may exist to provide an additional layer of protection from aberrant Ca^2+^ steady-state levels. This can be mediated by factors associated with SOCE, pumps that clear cytosolic Ca^2+^, or an increase in the cell-buffering capacity (92–95).

Our study shows for the first time, to the best of our knowledge, that SARAF is an essential contributor to the dysregulation of general and PVN-regulated metabolic states. Moreover, it is a novel model for age-dependent sarcopenic obesity, which is not dependent on feeding or accompanied by diabetes. Pharmacological targeting of the SARAF signaling pathway may provide a novel approach for treating sarcopenic obesity.

## Materials and methods

### Mice

The SARAF conditional KO strain used for this research project was generated from KOMP ES cell line *Saraf^tm1a(KOMP)Wtsi^*, RRID:MMRRC_061775-UCD; was obtained from the Mutant Mouse Resource and Research Center (MMRRC) at the University of California at Davis, an NIH-funded strain repository; and was donated to the MMRRC by The KOMP Repository (University of California, Davis), originating from Pieter de Jong, Kent Lloyd, William Skarnes, and Allan Bradley, Wellcome Sanger Institute. The ES cells were created by introducing a splice acceptor/reporter cassette containing a poly-A site into an endogenous intron upstream of a critical exon. The cassette includes the lacZ gene and the neomycin resistance gene surrounded by FLP sites and the critical (third) SARAF exon floxed by loxP sites (Fig. 1A). The following Jackson laboratory mice lines were used for crossing and generation of conditional mice lines: Gt(ROSA)26Sortm1(FLP1)Dym, Tg(Pgk1-cre)1Lni, B6.FVB(129X1) Tg(Sim1-cre)1Lowl/J. All general and spatially specific SARAF knocked-out mice were compared to their wild-type littermates.

All the animal procedures were approved by the Weizmann Institute Institutional Animal Care and Use Committee (IACUC).

### Genotyping

Genomic DNA was isolated from a tail biopsy using 25-mM NaOH and 0.2-mM disodium EDTA extraction buffer (PH12) incubated for 1 h at 95°C, the extract was neutralized using 40-mM Tris–HCl neutralization buffer (PH5), and PCR identified mouse genotypes. Two PCR primers were synthesized to detect the intact *Cre* gene; three PCR primers were synthesized to detect the KOMP, WT, and loxP inserted SARAF gene, and four PCR primers were used to detect WT, KO, and HET mice in the SARAF^fl/fl^PGK-Cre line (see Table S1). The PCR conditions were 95°C denaturing, 60°C (for SARAF), 55°C (for Cre), 65°C (for SARAF^fl/fl^PGKCre) annealing, and 72°C extensions for 35 cycles using Taq polymerase and a DNA Thermal Cycler from Bio-Rad. The PCR products were resolved by electrophoresis in 1% agarose gel.

### Mouse embryonic fibroblasts

E14.5 embryos were dissected, and their trunks were extracted into cold PBS. The trunks were finely minced using a razor until they were pipettable. Cells were suspended in 3-ml trypsin-EDTA 0.05%, at 37°C for 10 min. Cells were flushed through a 21-g (3 ml) syringe three times and double serum levels to block the trypsin activity. Next, cells were centrifuged at 1,800 rpm for 5 min, and the pellet was re-suspended in 8 ml of MEF media, containing 10% fetal calf serum, 2% Penstrep, 1% L-glutamine, and 1% sodium pyruvate. Cells from each embryo were plated on a 100-mm dish coated with 0.1% gelatin. Cells were split 1:4 and frozen when they reached confluence.

### Primary hepatocyte

Mouse hepatocytes were isolated from 8 to 12-week-old male mice. Mice were anesthetized using ketamine and xylazine; the liver was exposed, perfused in two steps, and washed, and a digestion step was done by Liberase research-grade (Roche Diagnostics). Cells were centrifuged in Percoll (GE Healthcare) gradient and seeded on collagen type 1 (Sigma)-coated plates with HBM basal medium + HCM single Quotes (Lonza) media containing 1% FBS for 2.5–3 h, later washed with non-serum-containing media, and used for experiments for up to 24 h.

### Primary hippocampal cultures

Cultures were prepared from E18-P0 mouse pups, and pups were decapitated, their brains removed, and the hippocampi dissected free and placed in chilled (4°C), oxygenated Leibovitz L15 medium (Gibco, Gaithersburg, MD, USA). The hippocampi were dissociated mechanically, and cells were plated in a 24-well plate, onto round coverslips coated with L-polylysine. Glia were plated 14 days beforehand and grown in 10% FBS and glutamate. Cultures grew in a medium containing 5% HS, 5% FBS, and B27 in an incubator at 37° C with 5% CO_2_. Cells were imaged 13–17 days after plating (Fig. S6).

### Behavioral assays

All behavioral assays were performed during the dark period (8 AM–8 PM) in a reverse cycle room. Before each experiment, mice were habituated to the test room for 2 h.

#### Morris water maze

For the acquisition phase, mice were subjected to four trials per day with an interval of 15 min, for 7 consecutive days. In each trial, the mice were required to find a hidden platform located 1 cm below the water surface in a 120-cm-diameter circular pool. In the testing room, only distal visual–spatial cues for locating the hidden platform were available. The escape latency in each trial was recorded up to 90 s. Each mouse was allowed to remain on the platform for 15 s and then was then removed from the maze. If the mouse did not find the platform within the 90 s, it was manually placed on it for 15 s. Memory was assessed 24 h after the last trial. The escape platform was removed, mice were allowed to search for it for 1 min, and the time spent in the different quadrants of the pool was recorded using a VideoMot2 automated tracking system (TSE Systems).

#### Y-maze

The maze contains three arms at 120° from each other. The mouse underwent training and test on the same day. During training, one arm was closed. During the test, the mouse starts at the end of one arm and then chooses between the other two arms. The amount of time and duration spent in the closed arm is measured to demonstrate learning and memory.

#### Dark–light transfer test

The test consists of a polyvinyl chloride box divided into a dark black compartment (14 × 27 × 26 cm) and a white 1050-lx illuminated light compartment (30 × 27 × 26 cm) connected by a small passage. Mice were placed in the dark compartment to initiate a 5-min test session. The time spent in the light compartment and the number of entries to the light compartment were measured.

#### Acoustic startle response

Mice were placed in a small Plexiglas mesh cage on top of a vibration-sensitive platform in a sound-attenuated, ventilated chamber. A high-precision sensor integrated into the measuring platform detected the movement. Two high-frequency loudspeakers inside the chamber produced all the audio stimuli. The ASR session began with 5-min acclimatization to white background noise (65 dB) maintained throughout the session. Thirty-two startle stimuli (120 dB, 40-ms duration with a randomly varying ITI of 12–30 s) were presented. The reaction time and latency to peak startle amplitude were measured.

### Immunohistochemistry

The mice were anesthetized and perfused with 1% PFA in PBS. Brains were carefully removed and fixed overnight in 30% sucrose and 1% PFA in PBS. The following day 30-µm coronal slices were prepared using a sliding microtome and stored in PBS 0.01% Na-Azid.

For X-gal staining, the 30-µm slices were incubated in PBS containing 1% glutaraldehyde for 4 min and washed three times in PBS. Slices were incubated overnight in X-gal staining solution containing 3-mM K_3_Fe(CN)_6_, 3-mM K_4_Fe(CN)_6_, 1.3-mM MgCl_2_, 0.02% NPO_4_, 0.01% NaDOC, and 1-mg/ml X-gal in PBS, filtered through a 0.45-µM filter. The next day slices were washed three times in PBS and mounted on slides.

For immunohistochemistry, the following primary antibodies were used: rabbit antibeta-galactosidase (1:1,000; Cappel) and goat antidoublecortin (1:100; Santa Cruz). Slices/cells were first incubated for 1.5 h at room temperature in a blocking solution containing 20% normal horse serum (NHS) and 0.3% triton ×100 in PBS, followed by 48–72-h incubation at 4°C in a primary antibody solution containing 2% NHS and 0.3% triton ×100 in PBS. The slices were then washed with PBS and incubated with biotin-conjugated goat antirabbit (1:200; Jackson) in 2% NHS-PBS for 1.5 h at room temperature. Finally, slices were incubated for 1 h at room temperature with secondary antibody FITS-conjugated donkey antigoat (1:200; Jackson) and Cy3-streptavidin (1:200; Jackson). Slices were then washed with PBS and incubated for 10 min with Hoechst (1:2,000), washed, and mounted for fluorescence imaging.

Cells were fixed with 4% PFA in PBS for 15 min, washed, and permeabilized using 0.2% triton ×100 in PBS for 30 min. Cells were blocked for 1.5 h, with 5% horse serum in PBS. The primary rabbit anti Ki67 (1:200, Abcam) antibody was incubated overnight. Secondary goat antirabbit cy3 (1:200, Jackson) antibody was incubated for 30 min. Cells were then washed with PBS and incubated for 10 min with Hoechst (1:2,000), washed, and mounted for fluorescence imaging.

#### EdU-proliferating cell staining

Mice were given 0.2-mg/ml EdU (CarboSynth) in drinking water for 2 weeks and then sacrificed. Brains were embedded in paraffin, and hippocampal slices were then used for immunostaining, using the Click-iT EdU imaging kit (Invitrogen). Staining was done according to manufacturer instructions.

### Rabbit polyclonal anti-SARAF antibody production

Rabbit polyclonal anti-SARAF antibody production was performed with the help of the Weizmann Institute core facilities and antibody engineering unit. Two NZW SPF rabbits (New Zealand white rabbits) were subcutaneously injected with SARAF's luminal domain (aa 30–164) fused to a 6×Histidine tag (41). The first injection was done with Freund's complete adjuvant (Difco 263810), and the second was given a week later with Freund's incomplete adjuvant (263910). Three boosters with an interval of 14 days were given IP in PBS, and serum was collected after each boost. The serums were purified for mouse IgG subclasses by affinity chromatography on protein-A Sepharose beads CL-4B (SPA-Sepharose, Pharmacia, Sweden). The best serum of the two was selected (according to western blotting), and additional purification steps were conducted as follows: rabbit serum was filtered using a 0.2-mM filter (Nalgene) and loaded onto the affinity HisTrap-NHS column. The column was washed with 10-CV PBS, 15-CV washing buffer, and an additional 10-CV PBS. The first elution step was performed with a glycine buffer (0.1-M glycine titrated to pH 2.3 with HCl). The second elution step was then done using the 5 CV of tetra-ethyl-ammonium (TEA) pH 11.5 (titrated with NaOH). The second fraction, eluted under basic conditions, had superior selectivity and specificity compared to the total serum and was used for Western blot analysis. All columns used are commercially available as prepacked media from GE Healthcare.

### Western blot

Adult mice spleens were placed in PBS and minced mechanically. The crude tissue debris were allowed to sediment by gravitation for 5 min, and the supernatant was collected and centrifuged at 2,800*g* for 5 min. The resulting pellet was homogenized in lysis buffer [50-mM Tris-Cl (pH 7.6), 150-mM NaCl, 0.5-mM EDTA, 1% IGEPAL (CA-630, Sigma), and protease inhibitor cocktail (Roche)], incubated on ice for 30 min and centrifuged at 17,000*g* for 10 min. The supernatant was collected into a new tube containing sample buffer and incubated at 95°C for 5 min. The resulting protein extract (10 μg) was separated using a 12% SDS–polyacrylamide gel electrophoresis (SDS-PAGE), transferred to nitrocellulose membranes, blocked, and treated overnight with a rabbit polyclonal anti-SARAF (1:10,000), in TBS-T (50-mM Tris, 150-mM NaCl, pH 7.4, 0.1% Tween20) with 1% BSA. After washing, the membranes were incubated with horseradish peroxidase-conjugated goat antirabbit IgG antibody (Jackson) in TBS-T with 1% skim milk and analyzed using an enhanced chemiluminescence (ECL) detection system (Bio-Rad). For re-blotting with anti-GAPDH (1:10,000), the membranes were washed, incubated in stripping buffer (62.5-mM Tris-Cl pH 6.8, 2% SDS, and 0.1-M 2-mercaptoethanol) at 50°C for 30 min, and then underwent the same blocking and antibody incubation protocol as above using antibodies for GAPDH.

### Corticosteroid blood measurements

Corticosterone was measured 5 h after the dark cycle began using the DetectX Corticosterone CLIA kit (Arbor assays). Five-μl tail blood samples from mice were collected before (basal), immediately after 15 min of restraint stress, and 30 and 75 min from stress initiation. The restraint stress was induced using a cut 50-ml plastic conical tube. Plasma samples were immediately centrifuged and stored at −80° C until assays for hormone measurement were conducted. Blood was analyzed according to the manufacturer's instructions.

### Micro-CT

Mice were anesthetized with isoflurane (3% for induction, 1–2% for maintenance) mixed with oxygen (1 l/min) and delivered through a nasal mask. Once anesthetized, the mice were placed in a head-holder to assure reproducible positioning inside the scanner. The set of mice was scanned using a micro-CT device TomoScope 30S Duo scanner (CT Imaging, Germany) equipped with two source-detector systems. The operation voltages of both tubes were 40 kV. The integration time of protocols was 90 ms (360 rotation) for 3-cm length, and axial images were obtained at an isotropic resolution of 80 μ. Due to the maximum length limit, to cover the entire mouse body, imaging was performed in two parts with the overlapping area, and then, all slices merged into one dataset representing the entire ROI. The radiation dose for each mouse was 2.2 Gy. Fat quantification analysis was performed using a CT analysis (Skyscan Bruker MicroCT) software (version 1.19).

### Calcium imaging

Cells were plated onto 24-mm L-polylysine-coated cover glass 4–24 h before the experiment. Before the experiment, the cover glass was mounted on an imaging chamber and washed with a 0/2-mM Ca^2+^ solution. Fura-2 AM loading of cells was performed for 30–45 min. Cytosolic Ca^2+^ levels were recorded from Fura-2 AM–loaded cells, excited at wavelengths of 340/20 and 380/20 nm, and imaged with 510/80-nm filters. For all single-cell imaging experiments, traces are of averaged responses from 10 to 50 cells.

Ringer's solution with or without CaCl_2_ or solutions with the following constituents: Ca^2+^-free solution contained HBSS-/-, 20-mM HEPES, 1-mM MgCl_2_, 0.5-mM EGTA, and 10-mM glucose calibrated to pH = 7. 2–5-mM Ca^2+^ solution contained the same except for the absence of EGTA and the addition of 2–5-mM CaCl_2_ (36, 41). Hepatocytes were placed in Ca^2+^-free media, and 20-μM BHQ was used to empty the internal stores. SOCE was measured upon the introduction of 2-mM Ca^2+^ to the extracellular solution. SOCE in hippocampal neurons was measured in the presence of TTX (1 μM), APV (10 μM), NBQX (2 μM), and nifedipine (50 μM) in 1.8-mM Ca^2+^.

### Murine metabolic studies

Indirect calorimetry, food, water intake, and locomotor activity were measured using the LabMaster system (TSE Systems, Bad Homburg, Germany). Data were collected after 48 h of adaptation from singly housed mice. Body composition was assessed using the Bruker minispec mq7.5 live mice analyzer.

### Running wheel

Mice were singly housed in standard cages equipped with a running wheel for 4 weeks (Columbus Instruments). Distances were recorded every 15 min from a counter attached to the wheel. The wheel circumference (111.76 cm) was converted to kilometers.

### Cellular respiration

Measurement of intact cellular respiration was performed using the Seahorse XF24 analyzer (Seahorse Bioscience Inc.) and the XF Cell Mito Stress Test Kit according to the manufacturer's instructions. Respiration was measured under basal conditions and in response to oligomycin (ATP synthase inhibitor; 0.5 μM) and the electron transport chain accelerator ionophore, FCCP (trifluorocarbonylcyanide phenylhydrazone; 1 μM), to measure the maximal OCR. Finally, respiration was stopped by adding the electron transport chain inhibitor Antimycin A (1 μM) (96).

### Glucose tolerance test

Mice were fasted for 5 h and subsequently given 2-g/kg glucose solution by i.p. injection. Blood glucose was determined at 0, 15, 30, 60, 90, and 120 min after the glucose challenge (FreeStyle Freedom Lite, Abbott).

### RNA sequencing

Total RNA was extracted from the indicated cell cultures using the RNeasy kit (QIAGEN). Then, RNA integrity was evaluated on a Bioanalyzer (Agilent 2100 Bioanalyzer), requiring a minimal RNA integrity number (RIN) of 8.5. Libraries were prepared according to Illumina's instructions accompanying the TruSeq RNA Sample Preparation Kit v2 (cat # RS-122–2001). According to the manufacturer's instructions, sequencing was carried out on Illumina HiSeq 2500v4 SR60, 20 million reads per sample.

Sequenced reads were mapped to the Mus musculus genome version GRCm38, using TopHat v2.0.10. Genes were identified using a .gtf obtained from Ensembl release 82. Per gene, reads were counted using HTSeq. Normalization of reading counts and *P*-values for differentially expressed genes were computed using DESeq2.

### Statistical and image analysis

Images were analyzed and quantified using the Fiji/ImageJ software. *P*-values were calculated using a Student's *t*-test for statistical comparisons of mean values using the GraphPad Prism software. Differences were regarded as significant for *P* < 0.05 (*) and highly significant for *P* < 0.01 (**) and *P* < 0.001(***). All data were checked for normality and displayed as mean ±S.E.M.

## Supplementary Material

pgad068_Supplementary_Data

## Data Availability

The data that support the findings of this study are available on request from the corresponding author, E.R. RNA seq data were deposited to GEO-GSE193354 https://www.ncbi.nlm.nih.gov/geo/query/acc.cgi?acc=GSE193354.
