# Supplementary material for: Store-operated Ca2+ entry regulatory factor alters murine metabolic state in an age-dependent manner via hypothalamic pathways
Source: PNAS Nexus. 2023 Mar 4;2(3):pgad068. doi: 10.1093/pnasnexus/pgad068 (PMC10062355; doi:10.1093/pnasnexus/pgad068)
Supplement: pgad068_Supplementary_Data [file pgad068_supplementary_data.docx]

Supplementary Material

**
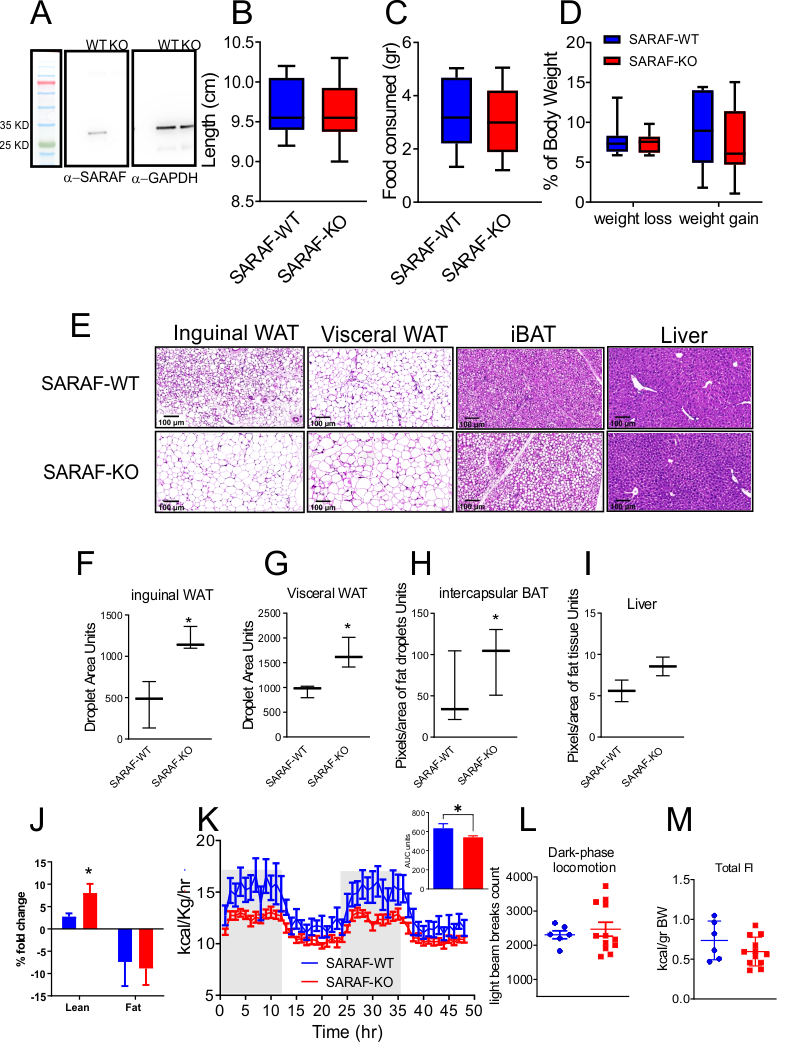
**

**Figure S1: Characterization of SARAF-KO metabolic phenotype. A**, Western blot of spleen extracts from SARAF-WT, and SARAF-KO mice. **B**, Linear growth measurement of the one-year-old mouse (SARAF-WT, n=10; SARAF-KO, n=6). **C**, food consumption 8 h refeed experiment, after 5 hours fasting during the active phase of three-month-old mice (SARAF-WT, n=7; SARAF-KO, n=9). **D**, Percentage of weight loss and gain following refeed experiment of three-month-old mice (SARAF-WT, n=7; SARAF-KO, n=9. **E**, histological sections of inguinal and visceral WAT, iBAT, and liver from three-month-old SARAF-WT, and SARAF-KO mice. Size bar- 100μm. **F-I**, quantification sections as outlined in E, Inguinal, and visceral WAT droplet size quantification, and iBAT and liver pixels of fat droplets/area quantification. **J**, Four-week voluntary wheel training effect percent fold change of lean/body weight and fat/body weight ratios. **K-M**, PhenoMaster calorimetry metabolic analysis of one-year-old mouse after four-week voluntary wheel training. **K**, Heat production over time (SARAF-WT, n=6; SARAF-KO, n=12). Insert AUC heat production. **L**, Dark-phase locomotion. **M**, Total food intake.

**
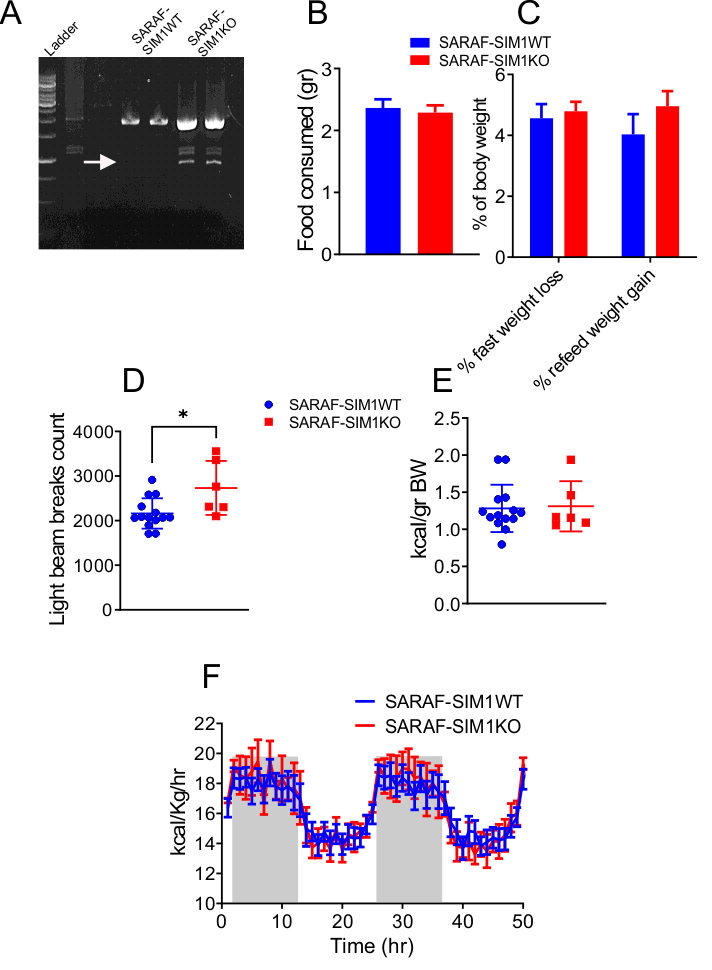
**

**Figure S2: Characterization of SARAF-SIM1KO metabolic phenotype.** **A** Tissue punch PCR validation of SARAF exon excision in the hypothalamus PVN, arrow indicates the product after the removal of exon 3. **B**, Food consumed after five hours fasting at eight hours refeed experiment in 1-years-old SARAF-SIM1WT and SARAF-SIM1KO mice. **C**, percentage of weight loss after five hours fasting, and gain after eight hours refeed experiment in 1-years old SARAF-SIM1WT (n=10) and SARAF-SIM1KO (n=6) mice. **D-F**, PhenoMaster calorimetry metabolic analysis of 3-month-old SARAF-SIM1WT and SARAF-SIM1KO mice **D,** Heat production over time (SARAF-SIM1WT, n=14; SARAF-SIM1KO, n=6). **E**, Dark-phase locomotion. **F**, Total food intake.

**
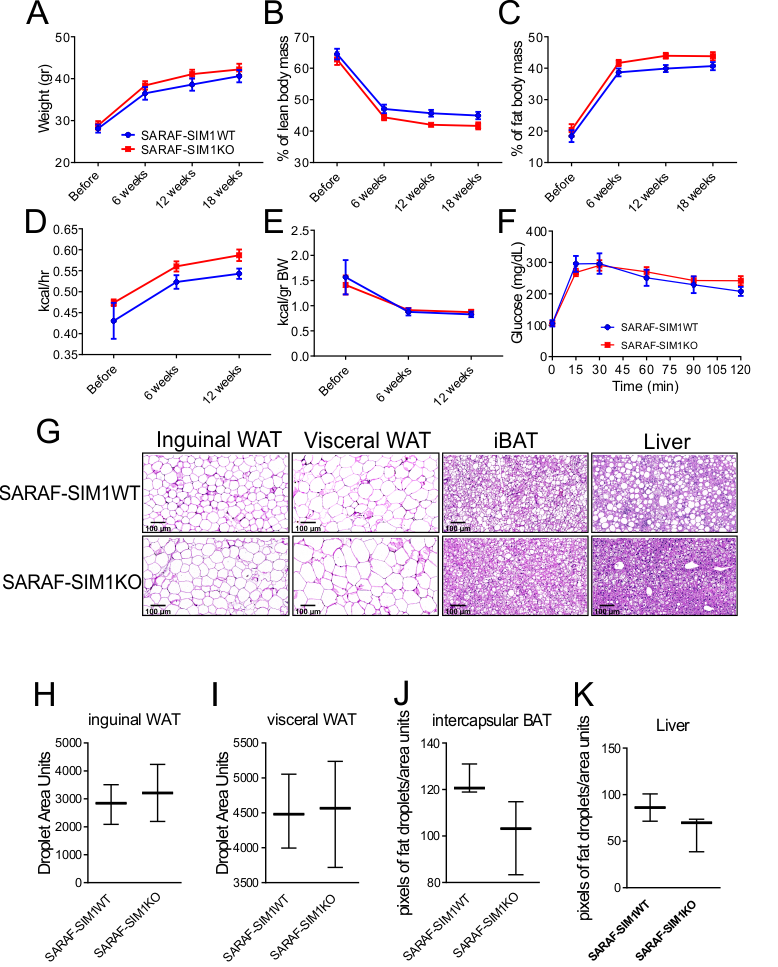
**

**Figure S3: Metabolic phenotype of western diet fed SARAF-SIM1WT and SARAF-SIM1KO. A** mice body weight (SARAF-SIM1WT, n=5; SARAF-SIM1KO, n=11), **B**, Percent lean mass change, and **C**, Percent change in body mass over eighteen weeks on a western diet. **D-E**, PhenoMaster calorimetry metabolic analysis during twelve weeks on western diet-fed mice. **D**, Heat production, **E**, Total food intake. **F**, Glucose tolerance test at twelve weeks on a western diet. **G**, Inguinal and visceral WAT, BAT, and liver H&E-stained tissue from mice after eighteen weeks western on fed diet. **H-K** fat droplets/area quantification of the sections as shown in G.

**
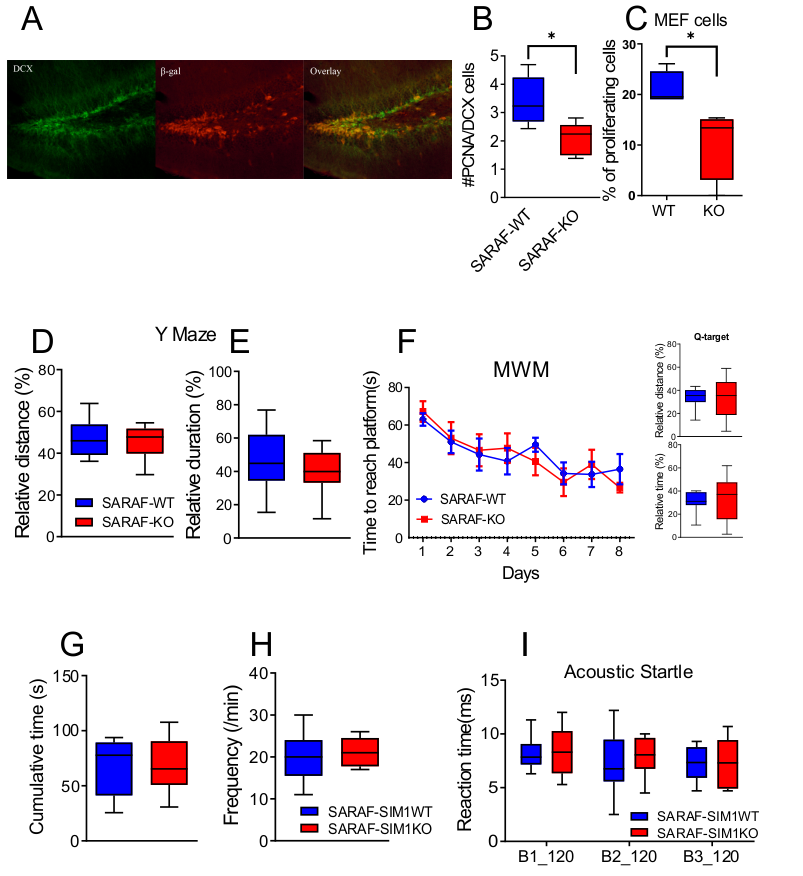
**

**Figure S4: Characterization of SARAF-KO and SARAF-SIM1KO mice memory and anxiety-related phenotypes. A**, Co-immunostaining of doublecortin (DCX) and β-gal and in a hippocampal section from *Saraf^tm1a(KOMP)Wtsi^* line. **B**, SARAF-WT, and SARAF-KO ventral hippocampal dentate gyrus proliferation quantification, number of PCNA positive cells out of DCX positive cells. **C**, Assessment of proliferation using Ki67 in MEF cells. **D-E,** Y-maze assay short memory test (SARAF-WT, n=11; SARAF-KO, n=10) and **F**, Morris Water Maze (MWM) long-term memory assay of the three-month-old SARAF-WT (n=7) and SARAF-KO (n=11) mice. **G-H** DLT. G, Time in light. F. The frequency of Visits in the lit section of three-month-old SARAF-SIM1WT and SARAF-SIM1KO mice. **I**, Acoustic startle response tests. Reaction time in 3 blocks of 120db stimuli of three-month-old SARAF-SIM1WT (n=14) and SARAF-SIM1KO (n=6) mice.

**
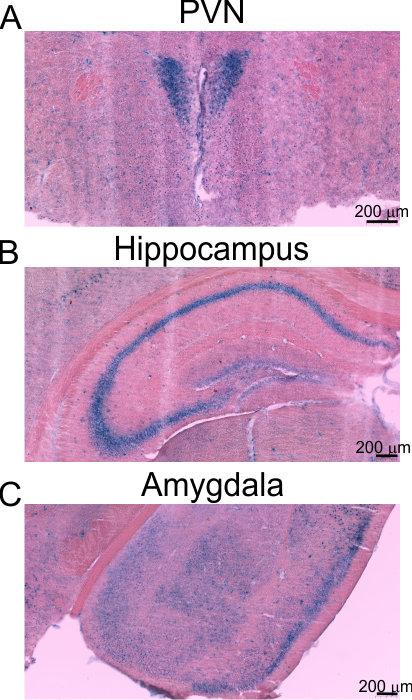
**

**Figure S5: High resolution images**. Images of the sections (as shown in Fig. 1B) of X-gal-stained coronal brain sections of KOMP cassette-inserted heterozygous mice, expressing β-gal at the sites of SARAF expression (scale bar 200μm).

**
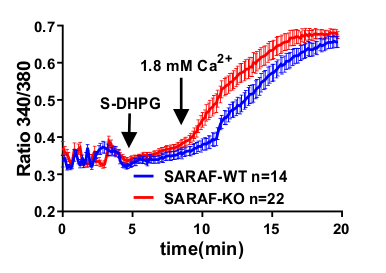
**

**Figure S6:** **SOCE in hippocampal neurons.** FURA-2 measurement of Ca2+ in primary cultured hippocampal neurons. Neurons were stimulated by 100 μM S-DHPG (mGluR1/5 agonist) to deplete Ca^2+^ from intracellular sores. 1.8 mM Ca^2+^ was replenished to reveal SOCE activity.

**
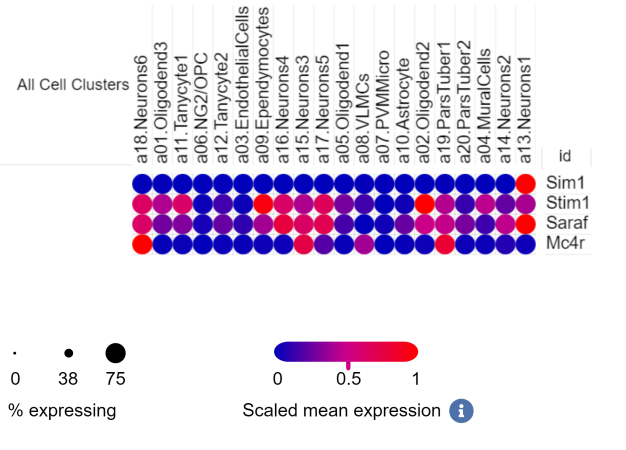
**

**Figure S7: Analysis of scRNAseq data obtained from various cell types in the arcuate hypothalamus and median eminence.** Deposited data was analyzed using the Single Cell RNAseq portal (63) Analysis was performed for SIM1, STIM1, SARAF, and MC4R.

**Table S1. Key resource.**

| **Reagent or resource** | **source** | **identifier** |
| --- | --- | --- |
| Antibodies | | |
|  | | |
| rabbit anti-beta-galactosidase | MP bio (Cappel) | Cat# 0855976 [AB_2334934](https://antibodyregistry.org/search.php?q=AB_2334934) |
| goat anti-doublecortin | Santa Cruz | Cat# sc-8066 [AB_2088494](https://antibodyregistry.org/search?q=sc-8066) |
| biotin-conjugated goat anti-rabbit | Jackson laboratories | Cat# 111-065-003 [AB_2337959](https://antibodyregistry.org/search?q=AB_2337959) |
| FITS -conjugated donkey anti-goat | Jackson laboratories | Cat# 705-095-147 [AB_2340401](https://antibodyregistry.org/search?q=AB_2340401) |
| Cy3-streptavidin | Jackson laboratories | Cat# 016-160-084 [AB_2337244](https://antibodyregistry.org/search?q=AB_2337244) |
| Rabbit anti Ki67 | Abcam | Cat# ab15580 [AB_443209](https://antibodyregistry.org/search.php?q=AB_443209) |
| Cy3 goat anti-rabbit | Jackson laboratories | Cat# 111-165-003 [AB_2338000](https://antibodyregistry.org/search?q=AB_2338000) |
| Rabbit polyclonal anti-SARAF | This paper | N/A |
| Peroxidase Goat Anti-Rabbit IgG | Jackson laboratories | Cat# 111-035-144 [AB_2307391](https://antibodyregistry.org/search?q=AB_2307391) |
| GADPH | Abcam | Cat# ab8245 |
| Chemicals, Peptides, and Recombinant Proteins | | |
|  | | |
| Hoechst 33342 | Life Technologies | Cat# H3570 |
| X-gal | Thermo Scientific | Cat#R0404 |
| Pierce™ 16% Formaldehyde | Thermo Scientific | Cat#28906 |
| DMEM - Dulbecco's Modified Eagle Medium Gibco™ | Thermo Scientific | Cat#41965-039 |
| Fetal bovine serum Gibco™ | Thermo Scientific | Cat#10270-106 |
| Dulbecco's Phosphate Buffered Saline | Biological Industries  (Sartorius) | Cat#02-023-1A |
| L-Glutamine solution | Biological industries  (Sartorius) | Cat#03-020-1B |
| Sodium pyruvate solution | Biological industries  (Sartorius) | Cat#03-042-1B |
| Pen-Strep solution | Biological industries  (Sartorius) | Cat#03-031-1B |
| Trypsin-EDTA solution | Biological industries  (Sartorius) | Cat#03-050-1B |
| Leibovitz L15 medium Gibco™ | Thermo Scientific | Cat#11415064 |
| MEM without L-glutamine Gibco™ | Thermo Scientific | Cat#21090-022 |
| Gentamicin | Sigma-Aldrich(Merck) | Cat#G-1272 |
| Glutamax Gibco™ | Thermo Scientific | Cat#35050-038 |
| Horse serum Gibco™ | Thermo Scientific | Cat#26050-047 |
| FUDR | Sigma-Aldrich(Merck) | Cat#F-0503 |
| Uridine | Sigma-Aldrich(Merck) | Cat#U-3750 |
| B27 supplements Gibco™ | Thermo Scientific | Cat#7504-010 |
| 0.1% Gelatine | Biological Industries | Cat#01-944-1B |
| Taq DNA Polymerase 2x Master Mix RED | Ampliqon | Cat# A190303 |
| Liberase research-grade | Roche Diagnostics | Cat#5401119001 |
| Percoll | Cytiva (GE Healthcare) | Cat#17089102 |
| Bovine serum albumin, fraction V | MP | Cat#160069 |
| Collagen, Type I solution from rat tail | Sigma-Aldrich(Merck) | Cat#C3867 |
| HBM ^TM^ basal medium | Lonza | Cat#CC-3199 |
| HCM^TM^ SingleQuots^TM^ Kit | Lonza | Cat#CC-4182 |
| Leibovitz L15 medium Gibco™ | Thermo Scientific | Cat#11415064 |
| Poly-L-lysine solution | Sigma-Aldrich(Merck) | Cat#P4707 |
| 2'-Deoxy-5-ethynyluridine (5-EdU) | CarboSynth | Cat#NE08701 |
| Freund’s complete adjuvant | Difco | Cat#263810 |
| Freund’s incomplete adjuvant | Difco | Cat#263910 |
| protein-A Sepharose beads CL-4B | Cytiva (GE Healthcare) | Cat#GE17-0780-01 |
| cOmplete™, Mini Protease Inhibitor Cocktail | Roche | Cat#11836153001 |
| CL47a complexiolyte solubilization buffer | Logopharm | N/A |
| Fura-2AM Calbiochem | Sigma-Aldrich(Merck) | Cat#108964-32-5 |
| carbachol | Sigma-Aldrich(Merck) | Cat#51-83-2 |
| Bradykinin acetate salt | Sigma-Aldrich(Merck) | Cat#B3259 |
| (S)-3,5-DHPG | Tocris | Cat#0805 |
| Vasopressin | Sigma-Aldrich(Merck) | Cat#113-79-1 |
| Thapsigargin | Alomone labs | Cat# T-650 |
| BHQ Calbiochem | Sigma-Aldrich(Merck) | Cat#286888 |
|  |  |  |
| Critical Commercial Assays | | |
|  |  |  |
| Click-iT EdU imaging kit | Invitrogen | Cat#C10339 |
| Pierce ^TM^ BCA kit | Thermo Fisher | Cat#23225 |
| WesternBright ECL HRP substrate | Advansta | Cat#K-12045-D20 |
| DetectX corticosterone CLIA kit | Arbor Assays | Cat#K014-C1 |
| XF Cell Mito Stress Test Kit | Agilent | Cat#103015-100 |
| RNeasy kit | QIAGEN | Cat#74104 |
| TruSeq RNA Sample Preparation Kit v2 | ILUMINA | Cat# RS-122–2001 |
|  |  |  |
| Deposited Data Submitted to GEO -GSE193354 https://www.ncbi.nlm.nih.gov/geo/query/acc.cgi?acc=GSE193354 | | |
|  | | |
| RNA-seq data | This paper |  |
|  |  |  |
| Experimental Models: Cell Lines | | |
|  | | |
| Saraf^tm1a^(KOMP)Wtsi ES clone | EUCOMM/KOMP | [061775-UCD](https://www.mmrrc.org/catalog/cellLineSDS.php?mmrrc_id=61775) |
| Mouse embryonic fibroblasts (MEFs) | This paper | N/A |
| Primary hepatocytes | This paper | N/A |
| Primary hippocampal cultures | This paper | N/A |
|  |  |  |
| Experimental Models: Organisms/Strains | | |
|  | | |
| SARAF^fl/fl^ | This paper | N/A |
| Gt(ROSA)26Sortm1(FLP1)Dym | The Jackson Laboratory | [#003946](https://www.jax.org/strain/003946) |
| Tg(Pgk1-cre)1Lni | The Jackson Laboratory | [#020811](https://www.jax.org/strain/020811) |
| B6.FVB(129X1) Tg (Sim1-cre)1Lowl/J | The Jackson Laboratory | [#006451](https://www.jax.org/strain/006451) |
| NZW SPF rabbits | ENVIGO | N/A |
|  |  |  |
| Oligonucleotides | | |
|  | | |
| Primer: Cre Forward: GGCCAGCTAAACATGCTTCA | This paper | N/A |
| Primer: Cre Reverse: ACACCAGAGACGGAAATCCATC | This paper | N/A |
| Primer: KO Forward: GGAACTTCGTCGAGATAACTTCGTATAGCATAC | This paper | N/A |
| Primer: WT Forward: ACAGGATGTACCTATGACAACAGTGGCAAT | This paper | N/A |
| Primer: Reverse WT: TAGACTCATGAATTTGAATGCCTGGCCC | This paper | N/A |
| Primer: PGK Forward 5546: CCCGACCCCTACAACACGTA | This paper | N/A |
| Primer: Reverse 5747: CACCACTCGACACTTCCGAT | This paper | N/A |
| Primer: Reverse 6290: CAGACTCGAGCGGTAGTCAAGTAGT | This paper | N/A |
|  |  |  |
| Software and Algorithms | | |
|  | | |
| VideoMot2 automated tracking system | TSE-Systems | <https://www.tse-systems.com/service/videomot2/> |
| PhenoMaster system | TSE-Systems | <https://www.tse-systems.com/service/phenomaster/> |
| FIJI/Image J | Schindelin et al., 2012(Schindelin et al., 2012) | <https://fiji.sc/> |
| Prism | GraphPad | <https://www.graphpad.com/scientific-software/prism/> |
| SlideViewer 2.5 | 3DHISTECH | <https://www.3dhistech.com/research/software-downloads/> |
| Inskape 1.1.1 | Inskape | <https://inkscape.org/> |
| Wave Controller Software 2.6 for XF24 analyzer | Seahorse Bioscience | <https://www.agilent.com/en/product/cell-analysis/real-time-cell-metabolic-analysis/xf-software/seahorse-wave-controller-software-2-6-1-740904> |
| Ingenuity® pathway analysis | QIAGEN | <https://digitalinsights.qiagen.com/products-overview/discovery-insights-portfolio/analysis-and-visualization/qiagen-ipa/> |
|  |  |  |
